# Temperature Prediction of Seasonal Frozen Subgrades Based on CEEMDAN-LSTM Hybrid Model

**DOI:** 10.3390/s22155742

**Published:** 2022-08-01

**Authors:** Liyue Chen, Xiao Liu, Chao Zeng, Xianzhi He, Fengguang Chen, Baoshan Zhu

**Affiliations:** 1 Badong National Observation and Research Station of Geohazards, China University of Geosciences, Wuhan 430074, China; liyuechen@cug.edu.cn (L.C.); zhubaoshan@cug.edu.cn (B.Z.); 2 China Communications Construction Company Second Highway Consultants Co., Ltd., Wuhan 430056, China; caozhen_shc@163.com (C.Z.); hexzh@126.com (X.H.); chenfengguang31@163.com (F.C.)

**Keywords:** seasonal frozen subgrade, temperature prediction, complete ensemble empirical mode decomposition with adaptive noise (CEEMDAN), long short-term memory (LSTM)

## Abstract

Improving the temperature prediction accuracy for subgrades in seasonally frozen regions will greatly help improve the understanding of subgrades’ thermal states. Due to the nonlinearity and non-stationarity of the temperature time series of subgrades, it is difficult for a single general neural network to accurately capture these two characteristics. Many hybrid models have been proposed to more accurately forecast the temperature time series. Among these hybrid models, the CEEMDAN-LSTM model is promising, thanks to the advantages of the long short-term memory (LSTM) artificial neural network, which is good at handling complex time series data, and its combination with the broad applicability of the complete ensemble empirical mode decomposition with adaptive noise (CEEMDAN) in the field of signal decomposition. In this study, by performing empirical mode decomposition (EMD), ensemble empirical mode decomposition (EEMD), and CEEMDAN on temperature time series, respectively, a hybrid dataset is formed with the corresponding time series of volumetric water content and frost heave, and finally, the CEEMDAN-LSTM model is created for prediction purposes. The results of the performance comparisons between multiple models show that the CEEMDAN-LSTM model has the best prediction performance compared to other decomposed LSTM models because the composition of the hybrid dataset improves predictive ability, and thus, it can better handle the nonlinearity and non-stationarity of the temperature time series data.

## 1. Introduction

Frozen ground occurs when the ground contains water, and the temperature of the ground is at or below 0 °C. Frozen ground can be divided into permafrost, seasonal frozen ground, and intermittently frozen ground. Permafrost usually remains at or below 0 °C for at least two years, while a layer of soil that freezes for more than 15 days per year is defined as seasonally frozen ground, and a layer of soil that freezes between one and 15 days a year is defined as intermittently frozen ground. China has the third largest permafrost area in the world, with seasonal frozen ground and permafrost accounting for 53.5% and 21.5% of China’s land area, respectively [1]. Seasonal frozen ground in China is distributed in the regions to the west of the Helan Mountain–Ailaoshan Mountain line, and the regions to the east of this line and to the north of the Qinling Mountains–Huaihe River line [2].

China’s One Belt One Road initiative has increased infrastructure development in the seasonal frozen region of Qinghai-Tibet, where several highway projects are planned [3,4,5]. The main hazard for subgrades in a seasonal frozen region is freeze-thaw damage, which is directly affected by the soil’s internal temperature. Therefore, the prediction of seasonal frozen soil temperatures has become a key issue and has received continuous attention from the academic community. Essentially, the temperature prediction problem belongs to time series forecasting. A time series is a data sequence arranged according to the chronological order of its occurrence. The main purpose of time series analysis is to forecast the future based on existing historical data. There are many classical time series analysis models, such as the autoregressive (AR) model, autoregressive moving average (ARMA) model, autoregressive conditional heteroskedasticity (ARCH) model, and the generalized autoregressive conditional heteroskedasticity (GARCH) model, and some improved algorithms have been widely used for prediction purposes [6,7,8,9,10]. However, these methods are often limited to linear and stationary time series forecasting. With the rapid development of information technology and the huge improvement of computer performance in the past two decades, many machine learning methods have been applied to the field of time series analysis, such as random forests (RF) [11], support vector machines (SVM) [12], Bayesian [13], extreme learning machines (ELM) [14], and Elman neural networks (ENN) [15]. Lin et al. [16] proposed a random forest-based model for extreme learning machine integration. Santamaría-Bonfil et al. [17] proposed a hybrid method for wind speed time series prediction based on support vector regression (SVR). Chen et al. [18] proposed a time series forecasting method based on a weighed least-squares support vector machine (LSSVM). Ticknor et al. [19] proposed a new Bayesian-regularized artificial neural network (BR-ANN) prediction method. Khellal et al. [20] proposed a method used to learn neural network features based entirely on an extreme learning machine (ELM). These aforementioned methods perform an essential role in time series forecasting with small and uniform data. However, when the data are large, nonstationary, or nonlinear, the prediction results are far from satisfactory [21].

In recent years, the research of artificial neural networks has made breakthrough progress. Deep learning models can handle many of the practical problems that are not easily solved by traditional methods. It has been shown that deep learning models can approximate nonlinear functions with arbitrary precision, as long as enough neurons are available. The recurrent neural network (RNN) model is one of the deep learning models with time series processing capability, which is often used for predictions of various time series. However, when using RNN models to solve long sequence problems, the issues of gradient disappearance and gradient explosion are prone to occur. To address this problem, Hochreiter and Schmidhuber [22] proposed long short-term memory (LSTM) as a variant of RNN. Since LSTM can learn the long-term dependence of data, it is widely used in natural language processing [23], image recognition [24], speech recognition [25], time series prediction [26], and other fields. Ma et al. [27] proposed a short-time traffic prediction model that effectively captures nonlinear traffic dynamics through an improved LSTM model. Hao et al. [28] improved the LSTM model for predicting the trajectory of walkers. Li et al. [29] proposed an air pollutant concentration prediction model that inherently considers spatiotemporal correlation using an improved LSTM model. However, there are few reports on the temperature time series prediction of seasonal frozen subgrades.

Due to the complex nonlinearity and non-stationarity of time series, a single prediction model sometimes gets stuck in local minima, resulting in suboptimal results. Therefore, more and more hybrid models have been proposed to obtain more accurate time series forecasting results. To maximize the use of the information contained in the history of time series, hybrid models are emerging, which generally combine two or more methods (modular unit). The mainstream hybrid models focus on the combination of data decomposition methods and forecasting models, where decomposition methods are an important preprocessing step in building these hybrid models. Empirical mode decomposition (EMD) is an adaptive decomposition algorithm for nonlinear nonstationary time series [30]. However, EMD cannot effectively decompose a nonstationary time series without sufficient extreme value points. In order to solve the mode mixing problem of EMD, Wu and Huang [31] proposed a white-noise-assisted data analysis method called ensemble empirical pattern decomposition (EEMD). Compared to EMD, EEMD eliminates the effect of mode mixing but still retains some noise in the intrinsic mode function (IMF). Therefore, a complete ensemble empirical mode decomposition with adaptive noise (CEEMDAN) algorithm is proposed by adding positive and negative auxiliary white noise pairs to the original time series data and introducing adaptive noise components, which not only maintains the ability to eliminate mode mixing and residual noise, but also has higher convergence performance with lower iterative cost [32].

Sha et al. [33] demonstrated that the input data pre-processed by CEEMDAN method can effectively improve the prediction performance of deep learning models. Hybrid models that combine these data decomposition methods with prediction models are widely used for time series analysis, such as the EEMD-ENN hybrid model used to predict annual runoff time series proposed by Zhang et al. [34]. Zheng et al. [35] proposed a hybrid SD-EMD-LSTM model for forecasting electrical loads. Lei et al. [36] proposed a hybrid model of EMD-SVR for predicting liquid level. Zhang et al. [37] proposed and validated the EEMD-LSTM hybrid model as a suitable model for temperature forecasting. Jiang et al. [38] proposed a CEEMDAN-FE-BILSTM hybrid model to predict PM2.5 concentration. Lin et al. [39] proposed a hybrid method combining CEEMDAN and ML-GRU (multi-layer gated recurrent unit) to accurately predict crude oil prices. Zhou et al. [40] proposed a hybrid model based on CEEMDAN, DL (deep learning), and ARIMA to predict short-term building energy consumption. Lin et al. [41] proposed a CEEMDAN-MLSTM hybrid model for reducing exchange rate risk in international trade.

This paper aims to more accurately predict the temperature of seasonal frozen stratification through the proposed CEEMDAN-LSTM model with a hybrid dataset. The prediction performance of the proposed model is compared with that of the other models through experimental results. The results show that the CEEMDAN decomposition method can better handle the nonlinearity and non-stationarity of temperature time series data, thus improving the prediction performance of neural network models. The models using hybrid datasets significantly improve the prediction performance compared to the models using single datasets, and the proposed CEEMDAN-LSTM model using hybrid datasets in all models has the best prediction performance. More details about the proposed method will be presented in the following sections.

## 2. Material and Methods

### 2.1. Case Study Area and Data Acquisition

**Figure 1:**
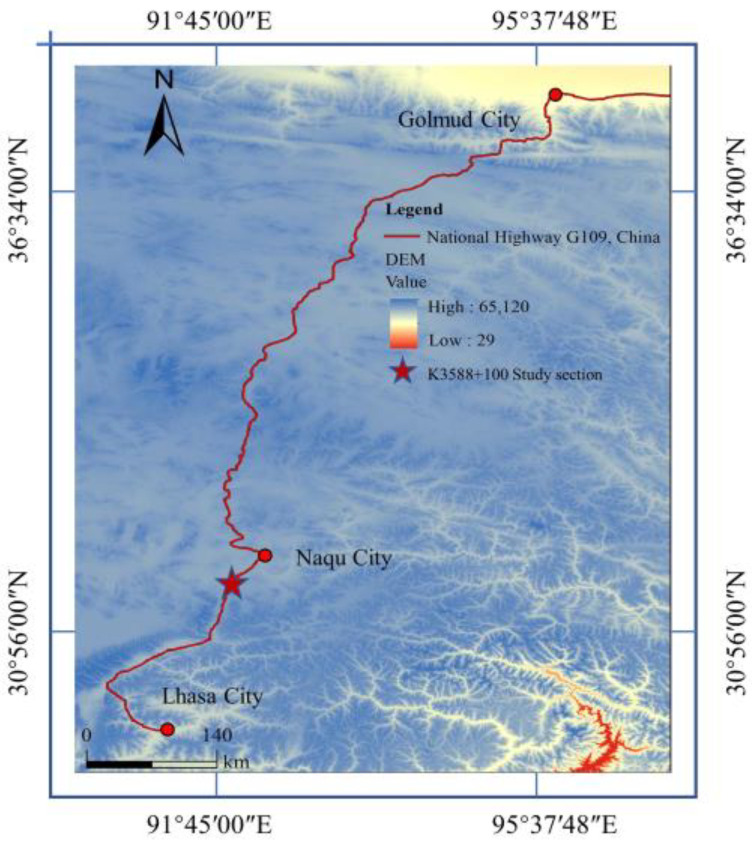
Study area.

The subgrade section to be researched in this paper was taken from the Golmud-Naqu highway, which is a part of China National Highway G109 connecting Beijing and Lhasa. It is located in Xiangmao Township (31°02′ N, 91°68′ E), Seni District, Naqu City, pile No. K3588 + 100, and the subgrade soil is mainly sandy soil. The location of section K3588 + 100 is shown in Figure 1. The area belongs to the plateau sub-cold semi-arid monsoon climate zone, with high terrain in the west and low terrain in the east. The elevation is between 3800–4500 m, with an average of about 4100 m. As a result of its high altitude, it suffers from a lack of heat and a harsh, arid climate. The annual average temperature is −2.2 °C, the annual relative humidity is 49%, the annual precipitation is 380 mm, and the annual sunshine hours are more than 2852 h.

**Figure 2:**
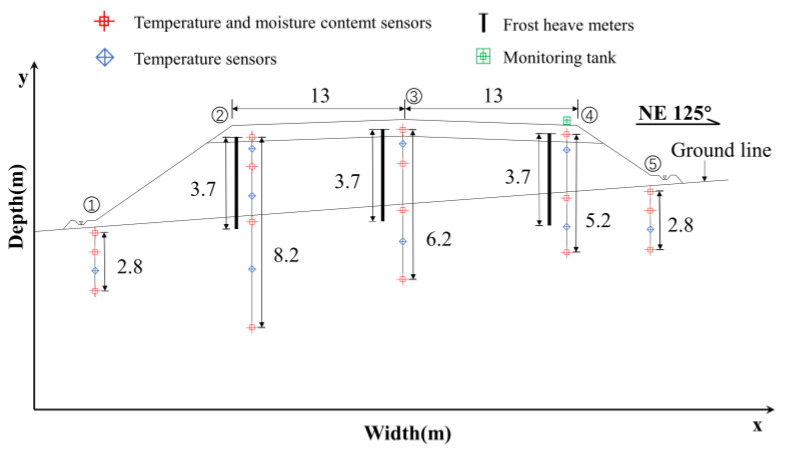
Study section layout.

**Figure 3:**
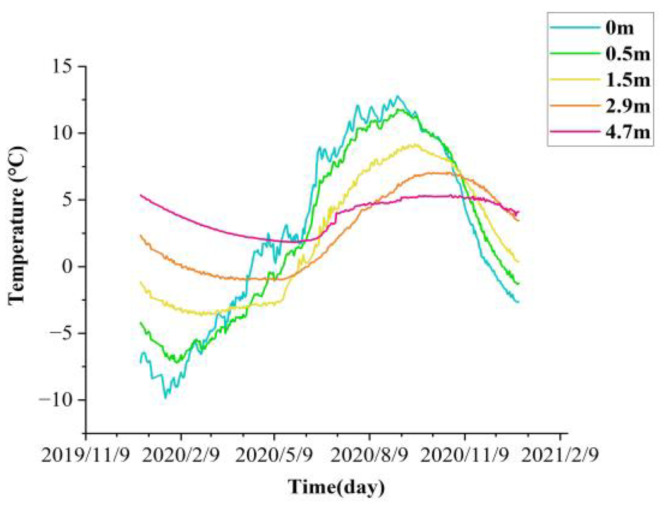
K3588 + 100 section right shoulder temperature monitoring curve.

The time series data were acquired through temperature sensors, volumetric water content sensors, and frost heave sensors deployed in the study area, the detailed study section layout is shown in Figure 2. In Figure 2, ①–⑤ are shown as the left foot of slope, left shoulder, roadbed median, right shoulder, and right foot of slope of the study section, respectively. The data duration is from 1 January 2020 to 31 December 2021, and the variation of temperature time-history at different depths of the right shoulder of section K3588 + 100 is shown in Figure 3.

### 2.2. Methods

#### 2.2.1. EMD and EEMD

Empirical mode decomposition (EMD) was proposed by Huang et al. in 1998 [30], Its main purpose is to decompose complex time series into a high-frequency part (i.e., the intrinsic mode function (IMF)) and a low-frequency part (i.e., the residual (*R*)). The decomposed IMF contains the features of the original time series at different time scales, and any time series can be decomposed into a finite number of IMF components with the residual.

The screening process for EMD is as follows:

Step 1: Find all local maxima and local minima of the original time series *S*(*t*), and then fit the upper envelope *U*(*t*) and lower envelope *L*(*t*) of the *S*(*t*) with the cubic spline interpolation function. The average of its upper and lower envelopes is *M*(*t*), as shown in Equation (1).

(1) $$M\left(t\right)=\frac{U\left(t\right)+L\left(t\right)}{2},$$

Step 2: Then, a new sequence *H*(*t*) is obtained by subtracting *M*(*t*) from the original time series *S*(*t*), as shown in Equation (2).

(2) H(t)=S(t)−M(t),

Step 3: If *M*(*t*) and *H*(*t*) satisfy termination criteria, then the first IMF as $c_{1}\left(t\right)=M\left(t\right)$, and the first residual *R* as $r_{1}\left(t\right)=H\left(t\right)$ are obtained. The termination criteria are: (i) *M*(*t*) approaches zero, (ii) the number of extreme points and zero crossing points in the *H*(*t*) differs by no more than 1.

Step 4: If the termination criteria are not satisfied, repeat steps 1 to 3 above for H(t) until $c_{1}\left(t\right)$ and $r_{1}\left(t\right)$ are obtained.

Step 5: Repeat steps 1 to 4 above for $r_{1}\left(t\right)$ until all IMFs and the residual are obtained. Thus, the original time series *S*(*t*) are decomposed as Equation (3):

(3) $$S\left(t\right)=\sum_{i=1}^{k}c_{i}\left(t\right)+r_{k}\left(t\right),$$

One of the shortcomings of EMD is mode mixing. When mode mixing occurs, a single IMF consists of different features of a time series signal, or features of a similar time series signal mixed in different IMFs. To alleviate this shortcoming, Huang et al. [31] proposed an improved algorithm called ensemble empirical modal decomposition (EEMD). It solves the mode-mixing problem in EMD by adding Gaussian white noise to the original time series signal. The specific steps are shown below:

Step 1: Add Gaussian white noise to the original time series, then obtain a new time series.

Step 2: Decompose the new time series to obtain each IMF component.

Step 3: Repeat steps 1 and 2 continuously, but each time using a different Gaussian white noise.

Step 4: Take the ensemble means of corresponding IMFs of the decompositions as the final result.

#### 2.2.2. CEEMDAN

Although EEMD overcomes the problem of mode mixing, the Gaussian white noise added to EEMD needs to be repeatedly averaged before it can be eliminated, which will take a large amount of computing time. Moreover, the reconstruction data still contains residual noise, and different realization of noise added to the time series could yield different numbers of modes. To solve the shortcomings of EEMD, Torres et al. [32] proposed a new decomposition method for time series signals, namely, complete ensemble empirical mode decomposition with adaptive noise (CEEMDAN). By adding positive and negative auxiliary white noise pairs to the original time series data and introducing adaptive noise components, the CEEMDAN algorithm not only maintains the capability of eliminating mode mixing and residual noise, but also has a lower cost for iterations and higher convergence performance. The specific decomposition algorithm is described as follows:

*S*(*t*) is the original time series, ${\bar{IMF}}_{k}\left(t\right)$ is the *k*th IMF obtained by CEEMDAN decomposition, $EMD_{j}\left(\;\right)$ represents the *j*th IMF obtained by EMD, $\varepsilon_{k}$ is a scalar coefficient to set the signal-to-noise ratio at each stage, and thus, to determine the standard deviation of the Gaussian white noise, $\omega^{i}\left(t\right)$ is the Gaussian white noise that meets the standard normal distribution. In this part of the calculation, S(t), ${\bar{IMF}}_{k}\left(t\right)$, $\omega^{i}\left(t\right)$, and r(t) represent a long vector of time series.

Step 1: By adding a white noise $\omega^{i}\left(t\right)\left(i\;=\;1,\;2,\;\ldots \ldots n\right)$ with signal-to-noise ratio $\varepsilon_{0}$ to the original time series $S^{i}\left(t\right)$, the *S*(*t*) obtained is used for the first decomposition, as shown in Equation (4). Where *t* represents the different time points, *i* represents the *i*th addition of white noise, and *n* represents the total number of added white noise.

(4) $$S^{i}\left(t\right)=S\left(t\right)+\varepsilon_{0}\omega^{i}\left(t\right),$$

Step 2: Use EMD to decompose $S^{i}\left(t\right)$ *n* times, and then obtain $IMF_{1}^{i}\left(t\right)$. The average value is then calculated according to Equation (5) to obtain the first IMF of CEEMDAN, the first residual $R_{1}\left(t\right)$ is obtained using Equation (6), and $EMD_{1}\left(\;\right)$ represents the first IMF obtained through EMD. In theory, since the average value of white noise is zero, the effect of white noise can be eliminated by calculating the average value.

(5) $${\bar{IMF}}_{1}\left(t\right)=\frac{1}{n}\sum_{i=1}^{n}IMF_{1}^{i}\left(t\right)=\frac{1}{n}EMD_{1}\left(S^{i}\left(t\right)\right),$$

(6) $$R_{1}\left(t\right)=S\left(t\right)-{\bar{IMF}}_{1}\left(t\right),$$

Step 3: The adaptive noise term is the first IMF obtained by EMD with the addition of white noise $\omega^{i}\left(t\right)$ with signal-to-noise ratio $\varepsilon_{1}$. Then the adaptive noise term is added to the first residual $R_{1}\left(t\right)$, obtaining a new time series. Subsequently, a new time series is decomposed to obtain the second IMF of CEEMDAN using Equation (7), the second residual $R_{2}\left(t\right)$ is obtained according Equation (8).

(7) $${\bar{IMF}}_{2}\left(t\right)=\frac{1}{n}\sum_{i=1}^{n}EMD_{1}\left(R_{1}\left(t\right)+\varepsilon_{1}EMD_{1}\left(\omega^{i}\left(t\right)\right)\right),$$

(8) $$R_{2}\left(t\right)=R_{1}\left(t\right)-{\bar{IMF}}_{2}\left(t\right),$$

Step 4: Repeat Step 3, the new time series is obtained by adding the new adaptive noise term to the residual term. Then decompose it to obtain the *k*th IMF of CEEMDAN, where $\omega^{i}\left(t\right),\left(i\;=\;1,\;2,\;\ldots n\right)$,$\;\varepsilon_{k},\left(k\;=\;2\;,\;3,\;\ldots K\right)$. The specific Equations (9) and (10) are as follows:

(9) $${\bar{IMF}}_{k}\left(t\right)=\frac{1}{n}\sum_{i=1}^{n}EMD_{1}\left(R_{k-1}\left(t\right)+\varepsilon_{k-1}EMD_{k-1}\left(\omega^{i}\left(t\right)\right)\right),$$

(10) $$R_{k}\left(t\right)=R_{k-1}\left(t\right)-{\bar{IMF}}_{k}\left(t\right),$$

Step 5: Finally, the CEEMDAN algorithm terminates when the residual term cannot continue the decomposition as it does not exceed two extreme points. At that time, the final residual R(t) is a distinct trend term. The full IMF and R(t) obtained are related to the original time series by the following Equation (11).

(11) $$S\left(t\right)=\sum_{k=1}^{K}{\bar{IMF}}_{k}\left(t\right)+R\left(t\right),$$

#### 2.2.3. LSTM

**Figure 4:**
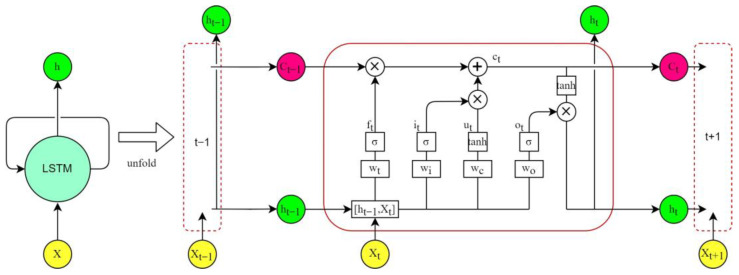
Structure of the unfolded LSTM cell. In the figure, ⊗ represents the Hadamard product, which is the multiplication of the corresponding elements of the matrix, ⊕ represents the sum of the corresponding elements of the matrix. *W* and *b* represent the weight matrix and bias matrix corresponding to the control gate, *σ* represents sigmoid activation, and tanh represents hyperbolic tangent activation.

Long short-term memory (LSTM) network is a special variant of recurrent neural networks (RNN) [22]. In traditional feedforward neural networks, information can only flow from the input layer to the hidden layer, and finally from one direction to the output layer. The main distinction between RNNs and feedforward neural networks is that RNNs have a recurrent cell to store the historical state of all past elements in the sequence [42,43]. However, when training an RNN model with the gradient descent method (usually used to train feedforward neural networks), the gradient may increase or decrease exponentially, which can cause the gradient to vanish or explode. If the gradient vanishes during the training process, the weights cannot be updated, which eventually leads to training failure. On the contrary, exploding gradients that are too large will drastically update network parameters, and, in extreme cases, can lead to erroneous results [44]. LSTM improved RNN by introducing the concepts of gates, and of applying memory cells to filter and process historical states and information instead of the recurrent cells of RNN. The basic structure of an unfolded single memory cell of an LSTM is shown in Figure 4. Each memory cell contains an input gate ($i_{t}$), a forget gate ($f_{t}$), and an output gate ($O_{t}$) to control the flow of information.

The input gate ($i_{t}$) determines how much input data needs to be stored in the cell state at the current moment (t) and the intermediate value ($u_{t}$) is used to update the cell state in the process of Equations (12) and (13).

(12) $$i_{t}=\sigma \left(W_{i}\cdot \left[h_{t-1},x_{t}\right]+b_{i}\right),$$

(13) $$u_{t}=\tan h\left(W_{c}\cdot \left[h_{t-1},x_{t}\right]+b_{C}\right),$$

The forget gate ($f_{t}$) determines how many cell states need to be retained from the previous moment (t−1) to the current moment (t), as shown in Equation (14).

(14) $$f_{t}=\sigma \left(W_{f}\cdot \left[h_{t-1},x_{t}\right]+b_{f}\right),$$

The cell state is updated from $C_{t-1}$ to $C_{t}$ by removing some of the old information and adding the filtered intermediate value $\left(u_{t}\right)$, as shown in Equation (15).

(15) $$C_{t}=f_{t}*C_{t-1}+i_{t}*u_{t},$$

The output gate ($O_{t}$) controls how much of the current cell state needs to be output to the new hidden state, as shown in Equations (16) and (17).

(16) $$O_{t}=\sigma \left(W_{O}\cdot \left[h_{t-1},x_{t}\right]+b_{O}\right),$$

(17) $$h_{t}=O_{t}*\tan h\left(C_{t}\right),$$

where in Equations (12)–(17), $x_{t}$ is the input at time t; $C_{t}$ and $C_{t-1}$ are the model output states at time t−1 and t, respectively; $h_{t-1}$ and $h_{t}$ are the outputs of the hidden layer at time t−1 and t, respectively; $u_{t}$ is the cell input state at time t. $f_{t}$, $i_{t}$ and $O_{t}$ are the outputs of the forget gate, input gate, and output gate at time t, respectively; $W_{f}$, $W_{i}$, $W_{O}$, and $W_{c}$ are the weights connecting $h_{t-1}$ and $x_{t}$ to the forget gate, input gate, output gate, and cell input, respectively; $b_{f}$, $b_{i}$, $b_{O}$, and $b_{C}$ are their corresponding bias terms.

In this study, the Adam optimization algorithm, which is a gradient descent method, was used. Because it has the good property of adjusting the learning rate adaptively, it is often used to calculate a weight matrix [45]. Adam combines the advantages of AdaGrad and RMSProp optimization algorithms with simple implementation, high computational efficiency, and less computing resources. Since the update of parameters in Adam is not affected by the gradient transformation, it is suitable for unstable or sparse gradients in very noisy datasets. Overfitting is a common phenomenon in LSTM, which results in a trained model with high accuracy on the training set, but low accuracy on the test set. Therefore, it is essential to prevent overfitting during training. Srivastava et al. [46] proposed a method, namely dropout, to prevent overfitting by dropping some random neurons from the network with a certain probability in each training process.

**Figure 5:**
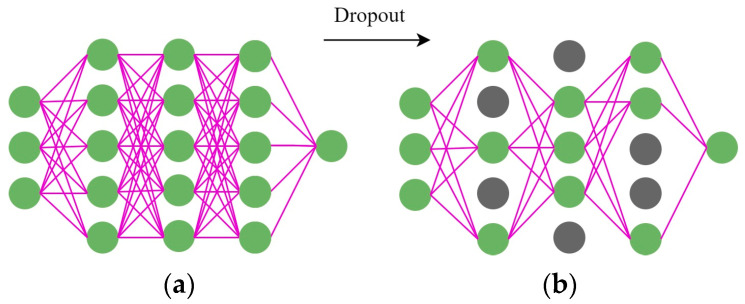
Flow chart of dropout method (Note: the green circles represent normal neurons, and the gray circles represent inactivated neurons). (**a**) the fully connected network; (**b**) the network after applying Dropout.

As shown in Figure 5, Figure 5a shows the fully connected network, and Figure 5b shows the network after applying the dropout method. Dropout can solve the overfitting problem by ignoring feature detectors to reduce the complex relationships between neurons and force the neural network model to learn better features.

## 3. Construction of CEEMDAN-LSTM Hybrid Prediction Model

### 3.1. Process of Constructing the Hybrid Prediction Model

**Figure 6:**
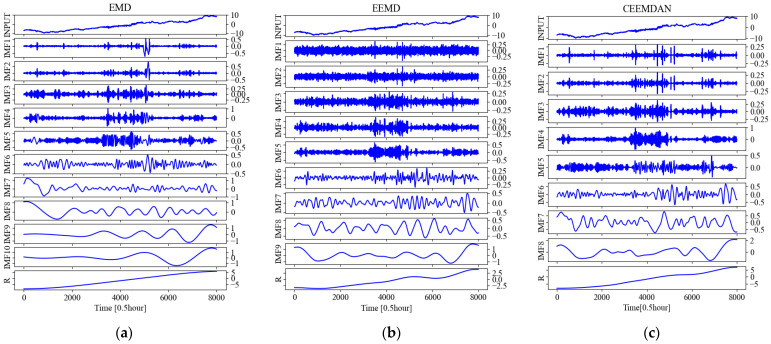
The results obtained after various decompositions for the temperature time series at 0.5 m below the subgrade surface of the right shoulder of section K3588 + 100 of China National Highway G109. (**a**) The IMFs and R obtained after EMD; (**b**) The IMFs and R obtained after EEMD; (**c**) The IMFs and R obtained after CEEMDAN.

Taking the temperature time series at 0.5 m below the subgrade surface of the right shoulder of section K3588 + 100 as an example, the IMFs and R (residual) obtained by various decomposing methods of EMD, EEND, and CEEMDAN are shown in Figure 6, respectively.

Previously, the process of CEEMDAN-LSTM for general prediction purposes could be divided into three steps. First, decompose the time series to be predicted into IMFs and a residual using CEEMDAN. Second, put each decomposed IMF as a single input vector into the LSTM neural network, and then obtain each corresponding IMF prediction. Third, add all the obtained IMF predictions to the residual term to obtain the final prediction value.

**Figure 7:**
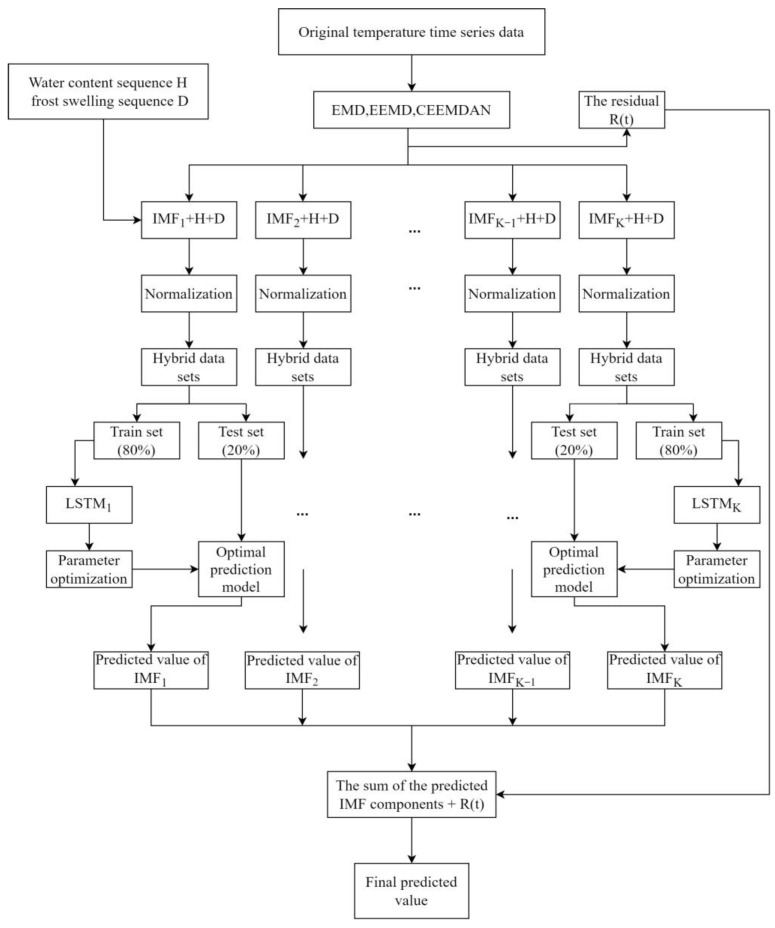
Flow chart of the experiment.

Considering that the temperature of the subgrade in the seasonal frozen region is mainly influenced by the freeze-thaw cycle of the subgrade soils, a new temperature strategy is proposed in this study. Both the measured volumetric water content and frost heave at the corresponding location are time series. Combine the three types of values (i.e., volumetric water content, frost heave, and IMFs) as a vector, and then construct a composite time series dataset. This dataset is used to train and test the LSTM model, and the specific steps are shown in Figure 7.

As shown in Figure 7, the EMD, EEMD, and CEEMDAN methods were used to decompose the original temperature time series data, respectively, and then the obtained IMF is combined with the volumetric water content time series (H) and the frost heave time series (D) to compose a hybrid dataset, respectively. 80% of the hybrid dataset is used as the train set and 20% as the test set. The train set is put into the lstm model to train to get the optimal prediction model, and then the test set is put into the optimal prediction model to get the IMFs predictions. Finally, all the IMF predictions and the residual term are added together to get the final prediction.

### 3.2. LSTM Neural Network Parameter Setting

**Figure 8:**
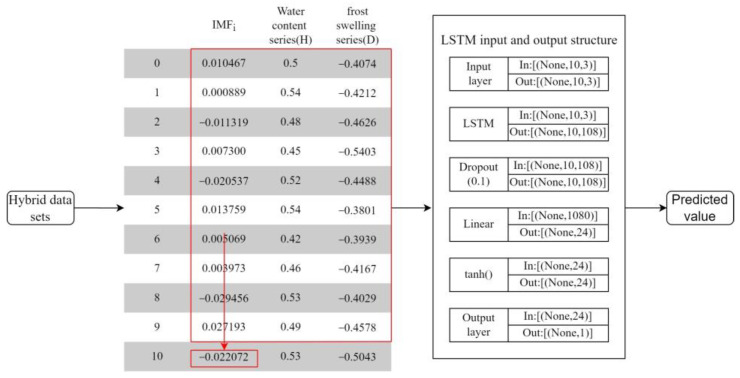
LSTM input and output schematic of *IMF_i_*.

As shown in Figure 8, ten continuous time series data are used in this study to predict the next time step data in the future, this means that it is a step ahead of the forecast, with the length of each time step being 30 min. Other hyperparameters are set as follows: LSTM with 3 hidden layers, 36 hidden layer neurons, 3 input features, Dropout regularization of 0.1, learning rate of 0.001, the final output layer is a linear layer, and the number of training epochs for each model is 200. The specific dimensional transformation from the data input to the predicted output is shown in Figure 8.

### 3.3. Model Evaluation Metrics

In this study, three evaluation metrics, namely root mean square error (*RMSE*), mean absolute error (*MAE*), and coefficient of determination (*R*^2^), were used to evaluate the relevant models. *RMSE* (for which a smaller value is better) is considered the most widely used error assessment method in point forecasting, and is generally more sensitive to large deviations between measured (actual) and predicted values. *MAE* (for which a smaller value is better) avoids the problem of errors canceling each other out, thus accurately reflecting the actual magnitude of forecast errors, while *R*^2^ (for which a bigger value is better) is used to estimate the degree of conformity between predicted and actual values. The specific formulas are as shown in Equations (18)–(20).

(18) $$RMSE=\sqrt{\frac{1}{n}\sum_{i=1}^{n}{\left(\hat{y}\left(i\right)-y\left(i\right)\right)}^{2}},$$

(19) $$MAE=\frac{1}{n}\sum_{i=1}^{n}\left|\hat{y}\left(i\right)-y\left(i\right)\right|,$$

(20) $$R^{2}\left(y,\hat{y}\right)=1-\frac{\sum_{i=1}^{n}{\left(\hat{y}\left(i\right)-y\left(i\right)\right)}^{2}}{\sum_{i=1}^{n}{\left(\hat{y}\left(i\right)-\bar{y}\right)}^{2}},$$

where $\hat{y}\left(i\right)$ is the predicted value, y(i) is the actual value, $\bar{y}$ represents the average of the actual value, and *n* is the total number of time series samples.

## 4. Results

### 4.1. Performance Comparison of Models Predictions before and after Decomposition

**Figure 9:**
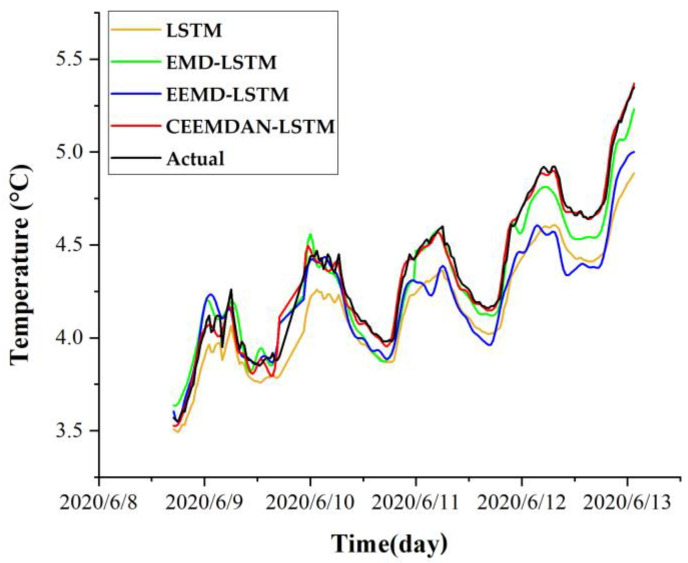
Comparison of prediction results of different models for 0.5 m under the right shoulder of the subgrade.

This study compares the prediction performance of the models before and after temperature time series decomposition to verify whether the effort of decomposition has a practical improvement on the prediction performance of the model. The EMD, EEMD, and CEEMDAN models are used as pre-processing to decompose the temperature time series, respectively, combined with an LSTM neural network to form three hybrid models (i.e., EMD-LSTM, EEMD-LSTM, and CEEMDAN-LSTM). The reconstructed hybrid datasets after the decomposition of temperature time series outperformed the undecomposed LSTM neural network model in prediction performance. The comparison of prediction performance of different models is shown in Table 1. The prediction accuracy of the EMD-LSTM, EEMD-LSTM, and CEEMDAN-LSTM models compared to the LSTM model improved by 57%, 9%, and 80% on *RMSE*, 59%, 18%, and 84% on *MAE*, and 8%, 4%, and 11% on *R*^2^, respectively. Compared to the LSTM, EMD-LSTM, and EEMD-LSTM models, the prediction accuracy of the CEEMDAN-LSTM model is 80%, 54%, and 78% improved over *RMSE*, 84%, 60%, and 80% improved over *MAE*, and 10%, 2%, and 7% improved over *R*^2^, respectively. These results show that the prediction performance after any decomposition by EMD, EEMD, or CEEMDAN is higher than that of the LSTM model without decomposition, indicating that the temperature time series is better predicted after decomposition. Among these models, the CEEMDAN-LSTM model obtains the highest prediction accuracy and performed significantly better than other models. A comparison of the prediction results of different models for 0.5 m under the right shoulder of the subgrade is shown in Figure 9.

### 4.2. Comparison of Prediction Results of LSTM Models for Single and Hybrid Datasets

**Figure 10:**
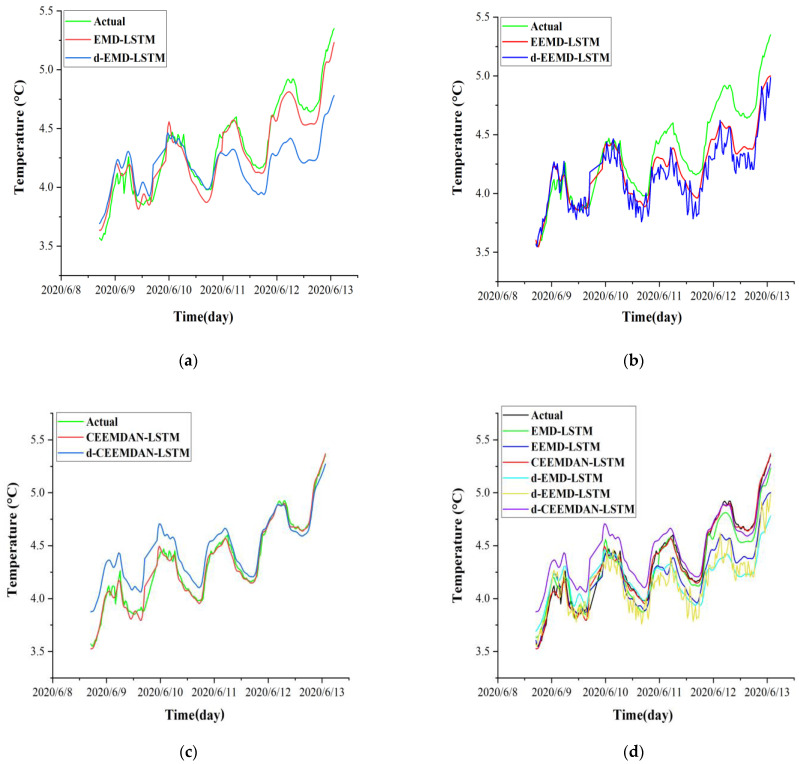
Comparison of prediction results of models with single dataset and hybrid dataset. (**a**) EMD-LSTM model (input hybrid dataset) versus d-EMD-LSTM model (input single dataset); (**b**) EEMD-LSTM model (input hybrid dataset) versus d-EMD-LSTM model (input single dataset); (**c**) CEEMDAN-LSTM model (input hybrid dataset) versus d-EMD-LSTM model (input single dataset); (**d**) Comparison of prediction results of all models.

In this study, the ability of the hybrid dataset to improve the model prediction performance was validated by comparing the prediction results of the single dataset and the hybrid dataset. The single dataset of the *i*th IMF obtained by EMD, EEMD, and CEEMDAN, respectively, was reconstructed into a hybrid dataset with volumetric water content time series (*H*) and frost heave time series (D) at corresponding depth. Subsequently, the hybrid dataset and single dataset were respectively input into the LSTM model for prediction. The prediction performances were compared in Table 2. To distinguish the names of each model, the following decomposed single-variable LSTM models are named d-EMD-LSTM, d-EEMD-LSTM, and d-CEEMDAN-LSTM. It can be seen that the LSTM prediction models that use the hybrid dataset are better than those LSTM prediction models that use the single variable dataset for all metrics. For example, the EMD-LSTM model improves the prediction accuracy of *RMSE*, *MAE*, and *R*^2^ by 65%, 65%, and 10%, compared to the d-EMD-LSTM model; the EEMD-LSTM model improves the prediction accuracy of *RMSE*, *MAE*, and *R*^2^ by 30%, 32%, and 3%, compared to the d-EEMD-LSTM model; and the CEEMDAN-LSTM improves the prediction accuracy of *RMSE*, *MAE*, and *R*^2^ by 75%, 77%, and 4%, compared to the d-CEEMDAN-LSTM model, respectively. Moreover, when the different decomposition methods are compared on different datasets, it can be seen that the prediction accuracy is the highest after CEEMDAN processing, and the CEEMDAN-LSTM model outperforms all the other models in terms of prediction accuracy. A Comparison of the prediction results of models with a single dataset and with a hybrid dataset is shown in Figure 10.

### 4.3. Comparison of Different Depth Prediction Models

**Figure 11:**
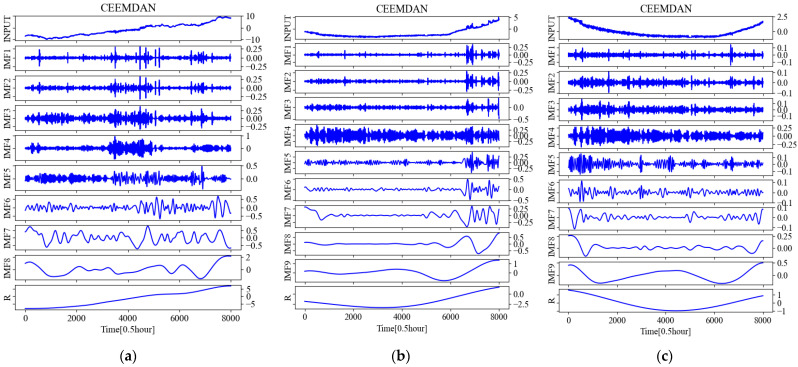
Results of temperature decomposed by CEEMDAN at different subgrade depths. (**a**) 0.5 m depth; (**b**) 1.5 m depth; (**c**) 2.9 m depths.

The data previously used in Section 4.1 and Section 4.2 is at a subgrade depth of 0.5 m. In order to verify the predictive performance of the CEEMDAN-LSTM model at different subgrade depths, and then to determine whether it can be applied to different depths of the subgrade, the monitored temperature time series at different depths were decomposed into EMD, EEMD, and CEEMDAN, then reconstructed into a hybrid dataset with the volumetric water content time series (*H*) and frost heave time series (D) at their corresponding depths, then put into the LSTM model for training and finally for prediction. Due to paper space limitations, only the figures of CEEMDAN decomposition are shown in Figure 11.

**Figure 12:**
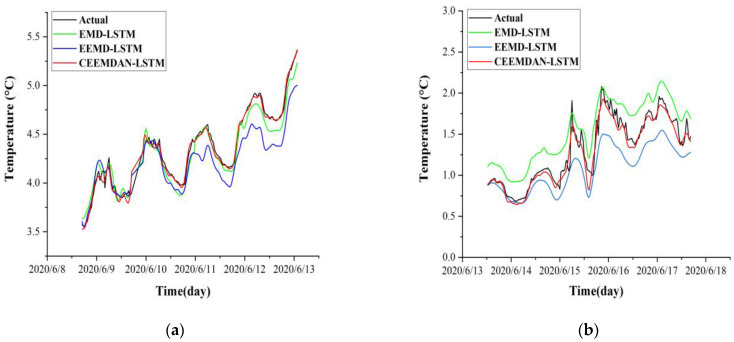

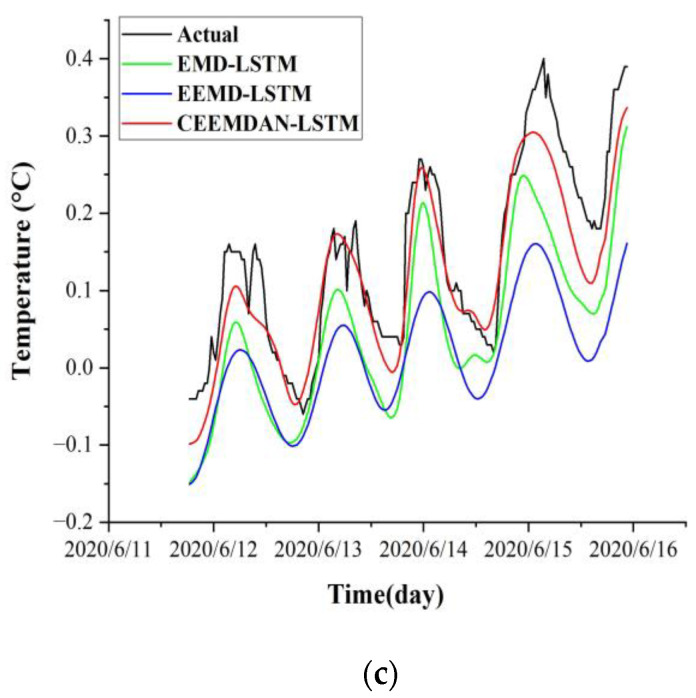
Comparison of model prediction results for different subgrade depths of the right shoulder. (**a**) 0.5 m depth; (**b**) 1.5 m depth; (**c**) 2.9 m depth.

The temperature time series were processed by EMD, EEMD, and CEEMDAN, respectively, then reconstructed into a hybrid dataset that contains the volumetric water content time series (*H*) and frost heave time series (D) of the corresponding depths. After that, the hybrid dataset was put into the LSTM model training, to get the optimal model, and to then make predictions. The specific prediction results are shown in Figure 12.

As shown in Figure 12, it can be seen that the prediction performance of the CEEMDAN-LSTM model is higher than that of the EMD-LSTM and EEMD-LSTM models at different subgrade depths, and the CEEMDAN-LSTM model does not decrease its prediction performance with the change of depth. However, as the depth of the stratum continues to increase, the temperature no longer changes significantly, and the temperature time series curve trend is nearly a smooth curve at this time. Then, the decomposition by EMD, EEMD, and CEEMDAN will no longer yield significant accuracy gains. This confirms that EMD-like methods are more suitable for time series with complex changes to obtain better decomposition. The detailed different performances are shown in Table 3.

As shown in Table 3, the prediction accuracy of the CEEMDAN-LSTM model at subgrade depth of 0.5 m is improved by 54% and 78% in *RMSE*, 60% and 80% in *MAE*, and 2% and 7% in *R*^2^, respectively, compared to the EMD-LSTM and EEMD-LSTM models. The prediction accuracy of the CEEMDAN-LSTM model at subgrade depth of 1.5 m is improved by 65% and 69% in *RMSE*, 72% and 73% in *MAE*, and 2% and 3% in *R*^2^, respectively, compared to the EMD-LSTM and EEMD-LSTM models. The prediction accuracy of the CEEMDAN-LSTM model at subgrade depth of 2.9 m is improved by 54% and 66% in *RMSE*, 59% and 70% in *MAE*, and 4% and 2% in *R*^2^, respectively, compared to the EMD-LSTM and EEMD-LSTM models.

## 5. Conclusions

Subgrade temperature prediction is of great significance for analyzing the thermal state of subgrades in seasonal frozen regions. In order to more accurately predict the subgrade temperature changes, a CEEMDAN-LSTM hybrid prediction model suitable for hybrid datasets is proposed. For various model comparison purposes, the original temperature time series were processed by EMD, EEMD, and CEEMDAN, respectively, and each IMF component was reconstructed into a hybrid dataset with the corresponding volumetric water content time series (*H*) and frost heave time series (D), and then the hybrid data were put into the LSTM neural network for training and prediction. Finally, the prediction results of different models were compared and analyzed, and the specific conclusions are as follows:

(1) By comparing the prediction performance of the original temperature time series model without decomposition and the models with different decompositions, it was found that the CEEMDAN-LSTM model among the hybrid models (i.e., EMD-LSTM, EEMD-LSTM, and CEEMDAN-LSTM) had the best prediction performance. Specifically, the prediction accuracy of the CEEMDAN-LSTM model was improved by 80%, 54%, and 78% in *RMSE* compared with the LSTM, EMD-LSTM, and EEMD-LSTM models, respectively. This means that the CEEMDAN decomposition method can better handle the nonlinearity and non-stationarity of the temperature time series data under the subgrade of seasonal frozen regions.
(2) Hybrid datasets significantly improved the prediction performance over single datasets, which is attributed to the strong extraction ability of LSTM neural networks for multidimensional features. It was found that models using hybrid datasets all outperformed models using single datasets, among which the CEEMDAN-LSTM model using hybrid datasets had the best prediction performance.
(3) In order to evaluate the prediction performance of models for different depths of the subgrade, predictions were applied to locations at subgrade depths of 0.5 m, 1.5 m, and 2.9 m. It was found that the CEEDMAN-LSTM model had the best prediction performance at all subgrade depths, and the performance did not decrease with depth. This means that the model can accurately predict the temperature inside the subgrade in seasonal frozen regions, which can provide reference and guidance for related research.

## Figures and Tables

**Table 1:** Comparison of prediction performance of different models.

Predictive Performance	LSTM	EMD-LSTM	EMD-LSTM	CEEMDAN-LSTM
*RMSE* (smaller is better)	0.210335	0.090355	0.191001	0.041966
*MAE* (smaller is better)	0.192146	0.079020	0.156670	0.031439
*R*^2^ (bigger is better)	0.893138	0.966392	0.925871	0.989248

**Table 2:** Comparison of prediction performance of models with single dataset and hybrid dataset.

Predictive Performance	d-EMD-LSTM	d-EEMD-LSTM	d-CEEMDAN-LSTM	EMD-LSTM	EEMD-LSTM	CEEMDAN-LSTM
*RMSE* (smaller is better)	0.286758	0.274303	0.168228	0.090355	0.191001	0.041966
*MAE* (smaller is better)	0.226918	0.230845	0.134943	0.079020	0.156670	0.031439
*R*^2^ (bigger is better)	0.876900	0.900513	0.955218	0.966392	0.925871	0.989248

**Table 3:** Comparison of prediction performance at different subgrade depths.

Depth under Subgrade	Predictive Performance	EMD-LSTM	EEMD-LSTM	CEEMDAN-LSTM
0.5 m	*RMSE*	0.090355	0.191001	0.041966
	*MAE*	0.079020	0.156670	0.031439
	*R* ^2^	0.966392	0.925871	0.989248
1.5 m	*RMSE*	0.245341	0.274970	0.086217
	*MAE*	0.229630	0.233995	0.064258
	*R* ^2^	0.947074	0.934600	0.964736
2.9 m	*RMSE*	0.102413	0.139584	0.047771
	*MAE*	0.092721	0.127279	0.038470
	*R* ^2^	0.925463	0.936328	0.960552

## Data Availability

Not applicable.
